# Magmatic evolution of a Cordilleran flare-up and its role in the creation of silicic crust

**DOI:** 10.1038/s41598-017-09015-5

**Published:** 2017-08-22

**Authors:** Kevin M. Ward, Jonathan R. Delph, George Zandt, Susan L. Beck, Mihai N. Ducea

**Affiliations:** 1 Department of Geology and Geophysics, University of Utah, Salt Lake City, Utah, 85721 USA; 2 Department of Earth Science, Rice University, Houston, Texas 77005 USA; 3 Department of Geosciences, University of Arizona, Tucson, Arizona 85721 USA; 4 Faculty of Geology and Geophysics, University of Bucharest, Bucharest, 010041 Romania

## Abstract

The role of magmatic processes as a significant mechanism for the generation of voluminous silicic crust and the development of Cordilleran plateaus remains a lingering question in part because of the inherent difficulty in quantifying plutonic volumes. Despite this difficulty, a growing body of independently measured plutonic-to-volcanic ratios suggests the volume of plutonic material in the crust related to Cordilleran magmatic systems is much larger than is previously expected. To better examine the role of crustal magmatic processes and its relationship to erupted material in Cordilleran systems, we present a continuous high-resolution crustal seismic velocity model for an ~800 km section of the active South American Cordillera (Puna Plateau). Although the plutonic-to-volcanic ratios we estimate vary along the length of the Puna Plateau, all ratios are larger than those previously reported (~30:1 compared to 5:1) implying that a significant volume of intermediate to silicic plutonic material is generated in the crust of the central South American Cordillera. Furthermore, as Cordilleran-type margins have been common since the onset of modern plate tectonics, our findings suggest that similar processes may have played a significant role in generating and/or modifying large volumes of continental crust, as observed in the continents today.

## Introduction

Continental crust covers ~40% of the Earth’s surface, comprises ~70% of the total volume of crust, and is (on average) intermediate in composition^1, 2^. However, the great majority of mantle-derived magmas are basaltic^3^ and fractional crystallization is not efficient enough to generate the volumes of silicic material observed in the continental crust unless delamination/recycling of its mafic/ultramafic cumulate counterpart is extremely efficient^4^. Thus, a long-standing question for Earth scientists is how such a large volume of intermediate-felsic composition continental material could be generated from an initial mantle-derived mafic melt. Most researchers agree that intermediate crustal formation or at least differentiation/diversification in modern and Phanerozoic systems takes place primarily in the upper plate of convergent margins^5^. A popular model to explain the compositional evolution of mafic material to more silicic compositions involves multiple stages of magma hybridization at different depths in the lithosphere of an arc (i.e., “hot zones”^6^). The size and volumes of these hot zones and thus their contribution to the production of silicic crust are not well known.

One approach to quantifying the magmatic volume in active convergent margins is through seismic imaging of the *in-situ* plutonic underpinnings below volcanic arcs and comparing the sizes of these bodies with their extruded equivalents (i.e., plutonic to volcanic, or P:V ratios). In general, quantifying well constrained P:V ratios is inherently difficult because the tectonic processes that exhume intrusive bodies rarely preserve their extrusive equivalents^7, 8^. Conversely, active magmatic systems that have well-preserved volcanic deposits require sophisticated geophysical or geochemical approaches to estimate their plutonic roots^9^. In this study, we use a recently developed multi-dataset inversion to examine the role of magmatic processes in the evolution and development of an ~800 km section (20.5°–28°S) of the active South American Cordilleran crust (the Puna Plateau). When placed in the context of existing geological and geophysical datasets, our seismic model reveals numerous large volume mid-crustal low-velocity zones that we interpret as the plutonic underpinnings associated with the voluminous silicic volcanics of the Puna Plateau.

## The Central Andean Plateau

**Figure 1:**
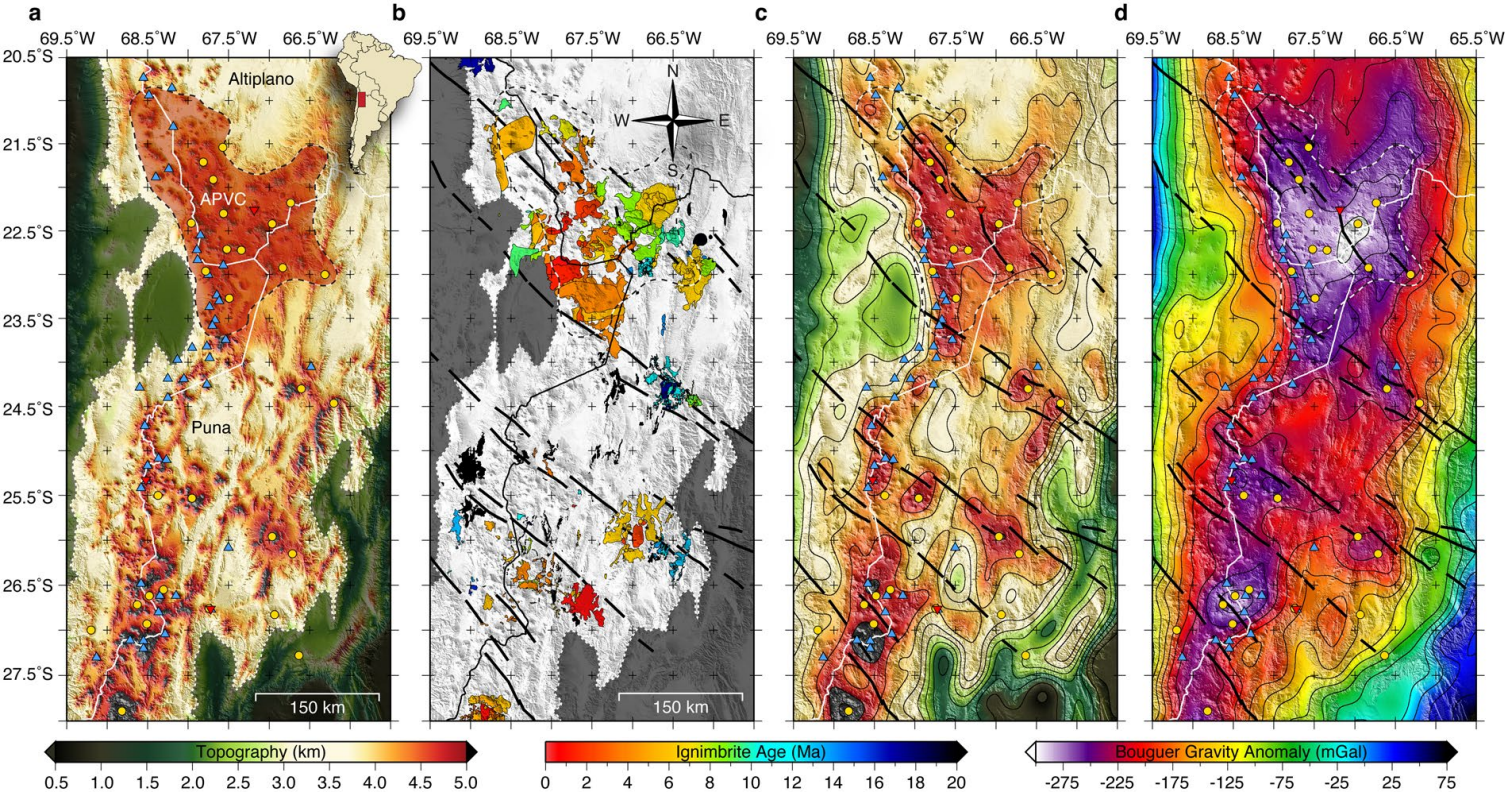
(**a**) Study area map within the Central Andean Plateau (CAP; <3 km masked) with the Altiplano-Puna Volcanic Complex (APVC) separating the Altiplano basin (northern CAP) and the high-relief Puna Plateau (southern CAP). Blue triangles show Holocene age volcanism of the Central Volcanic Zone (CVZ), gold circles show known ignimbrite eruption calderas, and inverted red triangles show INSAR measured vertical surface deformation centers^28^. (**b**) Distribution of Neogene ignimbrite deposits; black lines show the location of transverse lineaments^11, 59^. (**c**) Long-wavelength (>50 km) topography. (**d**) Long-wavelength (>50 km) Bouguer gravity anomalies^60^. These plots were made using the Generic Mapping Tool^61^, version 4.5.1 (ftp://ftp.soest.hawaii.edu/gmt).

Located along the active South American Cordillera, the Central Andean Plateau (CAP) is the largest orogenic plateau on Earth associated with long-lived subduction^10^. Forming the western margin of the CAP, the modern frontal volcanic arc (Western Cordillera) spans the entire length (14°–28°S) of the Central Volcanic Zone (CVZ). Along this section of the active arc, a large regional Neogene magmatic flare-up has erupted typical arc stratovolcanic edifices, low-volume rhyodacitic to rhyolitic ignimbrites, and voluminous monotonous dacitic ignimbrites^11^, as well as voluminous andesitic lavas. Separating the low-relief internally-drained Altiplano basin (northern CAP) and the high-relief Puna Plateau (southern CAP) into distinct morphotectonic provinces is the Altiplano-Puna Volcanic Complex (APVC; Fig. 1a), the location (21°–24°S) of a particularly intense and recent episode (11-1 Ma) of predominantly intermediate and silicic magmatism^12–14^ with lesser amounts of basalts^15^. The complicated magmatic history of the Puna Plateau may be linked to an episode of flat-slab subduction that swept southward across the Puna since ~18 Ma, where its modern location has resulted in volcanic quiescence that delineates the southern terminus of the CVZ at ~27.5°S^16, 17^. Evidence of lithospheric foundering after the slab resumed normal subduction further complicates the magmatic history of the Puna Plateau^18^.

The arid climate of the Puna Plateau has helped preserve > 18,000 km^3^ of silicic volcanic deposits^11^ (Fig. 1b) allowing the spatial-temporal evolution of ignimbrite emplacement to be mapped in detail^13, 14^. During the APVC ignimbrite flare-up episode (11-1 Ma), over 15,000 km^3^ of silicic volcanics were deposited, blanketing an area of ~70,000 (km^2^)^14^. For the first half of the flare-up (11-6 Ma), ~25–30% of the total erupted ignimbrite volume was spatially restricted to distinct arc and backarc locations before concentrating along the arc axis for the remainder of the flare-up^13^. Although smaller in erupted volume (~3,100 km^3^), the ignimbrites of the southern Puna (24°–28°S) resemble the spatial distribution mapped over the first-half of the APVC flare-up with distinct arc and backarc locations^11^. Magma mixing models constrain the crustal melt contribution of ignimbrites erupting through the thick crust (~70 km) of the Puna Plateau^19^ to range between 22–68% with a large amount of the Puna ignimbrites exhibiting a roughly 1:1 crust to mantle melt provenance^11, 19^.

Despite this wide spatial distribution, plateau ignimbrites display trace element ratios that indicate a clear arc affinity^11, 19^ and are dominantly calc-alkaline in composition, with some of the older eastern (backarc) deposits exhibiting mild to strongly peraluminous compositions indicating a greater crustal melt contribution^20–22^. However, disagreement persists in the petrologic community with regard to the arc origin of plateau magmatism in this case and elsewhere: some consider that these magmatic products are a natural extension and therefore part of the frontal arc since they have similar composition^14^, whereas others refer to these wide-spread manifestations of calc-alkaline magmatism as “Cordilleran interior” type magmatism^23^ which is distinct from the arc sensu-stricto and occurs on active plateaus.

**Figure 2:**
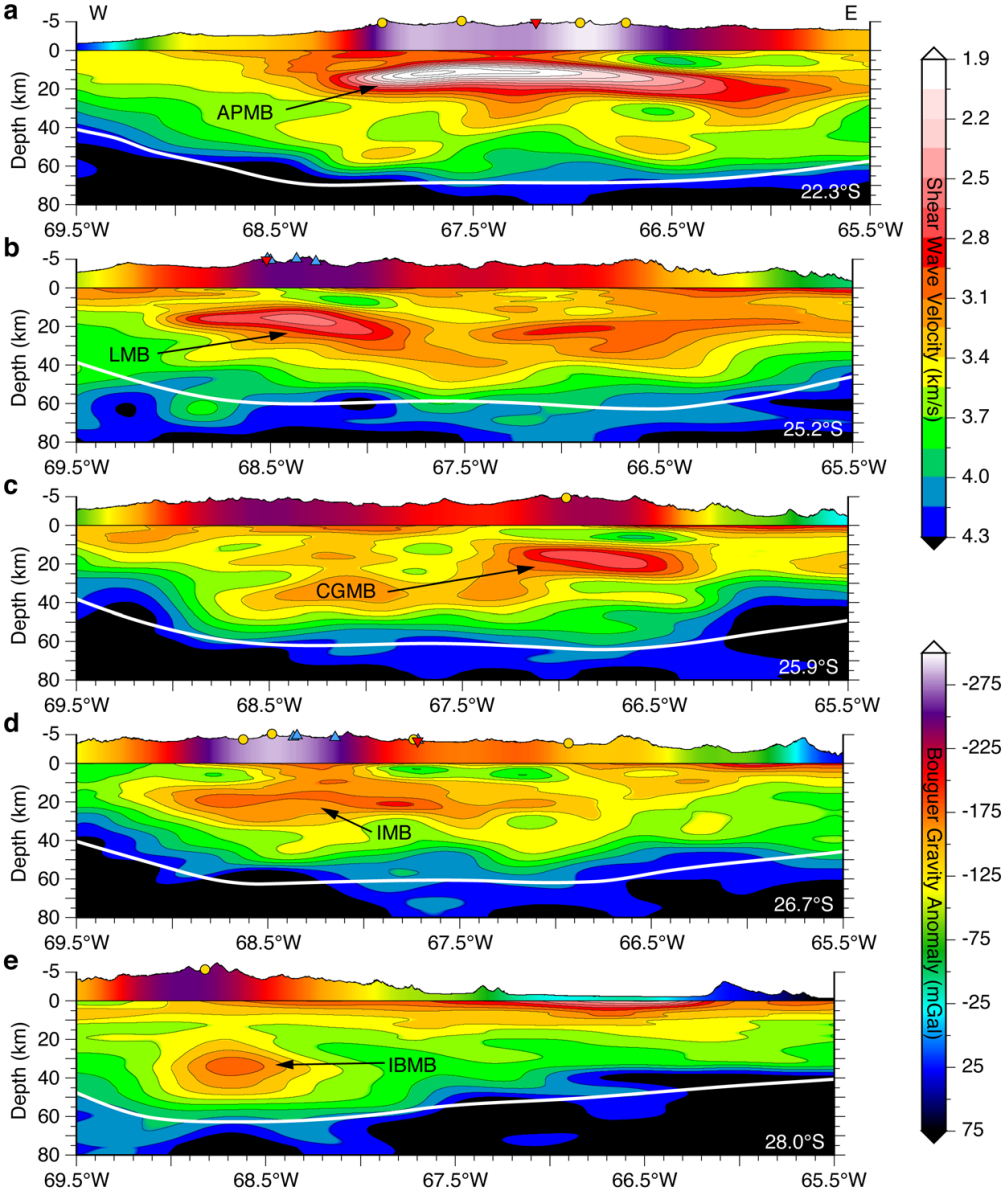
(**a–e**) West-east cross-sections through our shear-wave velocity model with the locations shown in the lower right corner. Blue triangles show Holocene age volcanism of the Central Volcanic Zone (CVZ), gold circles show known ignimbrite eruption calderas, inverted red triangles show INSAR measured vertical surface deformation centers, and white lines are the Moho^28, 62^. Vertically exaggerated (3:1) unfiltered topography is colored by the magnitude of the Bouguer gravity anomaly at that location^60^. The labels APMB (Altiplano-Puna Magma Body), LMB (Lazufre Magma Body), CGMB (Cerro Galan Magma Body), IMB (Incahuasi Magma Body), and IBMB (Incapillo-Bonete Magma Body) correspond to imaged magma reservoirs discussed in the text. These plots were made using the Generic Mapping Tool^61^, version 4.5.1 (ftp://ftp.soest.hawaii.edu/gmt).

Recent advances in crustal-scale seismic imaging have allowed the magmatic structure of active continental arcs to be imaged in unprecedented detail^24–26^. Building on local smaller-scale (<400 km) seismic studies in the APVC^24^ and southern Puna Plateau^26^, we follow the method presented by Delph *et al*.^27^ to generate a continuous regional-scale seismic velocity model (Vs) for the entire ~800 km length of the Puna Plateau. The nearly one-to-one spatial correlation we observe between the ignimbrite deposits (Fig. 1b), long-wavelength topography (Fig. 1c), long-wavelength Bouguer gravity anomalies (Fig. 1d), and mid-crustal low-velocity zones contained in our velocity model is consistent with a proto-plutonic interpretation. Five prominent low-velocity zones are highlighted in west-east cross-sections (Fig. 2) with correlations between Holocene volcanoes, known ignimbrite source calderas, and INSAR measured vertical surface deformation centers^28^ further supporting a magmatic/plutonic interpretation.

## Plutonic volumes and P:V ratios

Constraining the plutonic-to-volcanic relationship is an essential component of understanding the growth and development of continental crust and the evolution of Cordilleran systems^29^. Further complicating the enigmatic plutonic-to-volcanic relationship is the highly episodic nature of pluton emplacement, where magmatic flare-ups produce several orders of magnitude more magmatism when compared against magmatic lulls^30^. Until recently, limited and poorly constrained global averages of P:V ratios (~5:1^31^) have been used to investigate the role of magmatic processes in Cordilleran plateau development and formation, leading most researchers to dismiss magmatic addition as a significant contributor to the evolution of plateau formation in Cordilleran systems^10, 19, 32^.

Challenging the currently accepted role of magmatic processes in Cordilleran plateau formation is a small but robust collection of studies that suggest P:V ratios in Cordilleran systems may be as high as 20–75:1^8, 24, 30, 33, 34^. Several exhumed and titled Phanerozoic arc crustal sections, such as the nearly complete late Mesozoic Kohistan arc in Pakistan^35^ and the Ordovician Famatinian arc in Argentina^36^, along with at least a dozen others that are slightly less complete^5^, provide a geologic view into P:V ratios along arc sections. All of these example are composed almost exclusively of plutonic rocks of arc age, strongly suggesting that P:V ratios are in excess of 20:1^30^. Also, the Oligocene ignimbrite flare-up that accompanied the late evolutionary stages of the “Nevadaplano” plateau in western North America^37–39^ had P:V ratios similar to what recent studies in the central Andes^24, 33, 34^ have estimated based on the composition of mid-crustal exposures in highly extended domains of the Basin and Range^40^.

Typical crystallized plutonic compositions (granite/granodiorite/tonalitic) have an isotropic shear-wave velocity around 3.6 km/s at standard pressures and temperatures^41^; however, uncertainties in composition, temperature, and seismic anisotropy can reduce this to as low as ~2.9 km/s over the depth range our low-velocity zones occupy (see discussion in Ward *et al*.^24^). We therefore select the 2.9 km/s velocity contour as an upper limit when calculating the seismically imaged plutonic volume of our model, as velocities below 2.9 km/s almost certainly contain some partial melt. This approach yields inherently conservative plutonic volume estimates, as any crystallized plutonic bodies would be excluded from our estimates. Even with this restrictive definition, four distinct plutonic volumes are measured from north to south including (1), the Altiplano-Puna Magma Body (APMB; Fig. 2a) at ~520,000 km^3^, (2) the Lazufre Magma Body (LMB; Fig. 2b) at ~54,000 km^3^, (3) the Cerro Galan Magma Body (CGMB; Fig. 2c) at ~23,000 km^3^, and (4) the Incahuasi Magma Body (IMB; Fig. 2d) at ~12,000 km^3^. Velocities in the Incapillo-Bonete Magma Body (IBMB; Fig. 2e) are greater than 2.9 km/s and thus, not included in our plutonic volume estimates. However, velocities as low as 3.1 km/s are observed in the IBMB at ~40 km below the surface and at this depth, and such velocities probably require the presence of some partial melt. We note that although these crustal plutonic systems might better be described as crystal mush or magma reservoirs from a petrological viewpoint^42^, we retain the magma body nomenclature to avoid confusion with previous studies.

Having estimated the plutonic volumes associated with the major volcanic complexes of the Puna Plateau, we are able to calculate P:V ratios at progressively larger scales ranging from individual calderas to the entire Puna Plateau. At the individual caldera scale, the long-lived (2.56–5.60 Ma) Cerro Galan (Fig. 2c) silicic magmatic system has an erupted ignimbrite volume of 1,331 (km^3^)^43^ from which we calculate a ~17:1 P:V ratio. No volcanic volume estimates are available that can specifically be associated with the LMB (Fig. 2b) or IMB (Fig. 2d) magma reservoirs, thus the next largest scale we are able to calculate the P:V ratio for is the entire southern Puna Plateau (24°–28°S). An estimated 3,100 km^3^ of ignimbrites have erupted in the southern Puna^11^ which includes the LMB, CGMB, and IMB magma reservoirs resulting in a ~29:1 P:V ratio. At roughly the same spatial scale as the southern Puna, the APVC ignimbrite flare-up episode (11-1 Ma) erupted over 15,000 km^3^ of silicic volcanics accounting for nearly half of the ignimbrite volume deposited during the Central Andean regional Neogene magmatic flare-up^11, 14^ resulting in a ~35:1 P:V ratio. It is worth noting that Perkins *et al*.^34^ calculated the plutonic volume required to produce a ~1 km long-wavelength topographic dome observed in the APVC (Fig. 1c) using a buried load isostatic model, independently arriving at a plutonic volume (and P:V ratio) that is within ~7% of our seismically derived estimate. Finally, we are able to calculate a P:V ratio for the entire Puna Plateau, for which we obtain an estimate of ~34:1, which compares extremely well to recent estimates (>30:1) for the Late Cretaceous Cordilleran flare-up in the Sierra Nevada arc^30^ or the Ordovician arc at the Sierra de Valle Fértil titled exposure site^36^.

It is quite remarkable that the long-term P:V ratios measured at different scales along the Puna Plateau using completely independent methods have similar ratios (~30:1) estimated from the geologic record elsewhere. While it remains speculative that P:V ratios of ~30:1 are fundamental characteristics of Cordilleran magmatic flare-ups, it is clear that these ratios are much larger than those typicality invoked (~5:1) for these systems^31^, implying a much larger potential for the re-working and refinement of crust towards a more silicic composition in Cordilleran plateaus settings. Quantifying the surface uplift associated with the P:V ratios presented here for the Puna Plateau is beyond the scope of this study, but recent topographic modeling in the APVC suggests it might locally be quite large (~1 km)^34^.

## Magmatic evolution of a Cordilleran plateau

**Figure 3:**
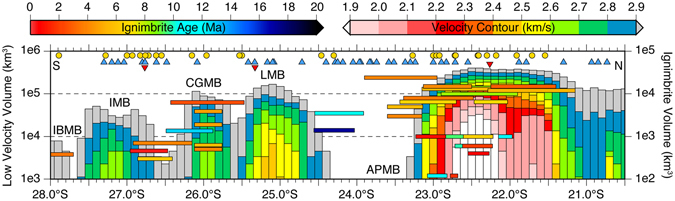
Along-strike spatial distribution of individual dated ignimbrite volumes (horizontal bars with volumes shown on right vertical axis; Freymuth *et al*.^11^ and references therein), Holocene age volcanism (blue triangles), known ignimbrite eruption calderas (gold circles), INSAR measured vertical surface deformation centers^28^ (inverted red triangles), compared against our seismic volumes enclosed by different velocity contours in 0.1° bins (with volumes shown on left vertical axis). The labels APMB (Altiplano-Puna Magma Body), LMB (Lazufre Magma Body), CGMB (Cerro Galan Magma Body), IMB (Incahuasi Magma Body), and IBMB (Incapillo-Bonete Magma Body) correspond to imaged magma reservoirs discussed in the text. This plot was made using the Generic Mapping Tool^61^, version 4.5.1 (ftp://ftp.soest.hawaii.edu/gmt).

The main driving mechanism responsible for the magmatic flare-up in the Puna Plateau is thought to be the southward passage of flat-slab subduction, subsequent re-steepening of the slab, and associated lithospheric delamination^44, 45^, potentially analogous to the evolution of western North American during the Laramide Orogeny^46^. Numerous systematic relationships observed in our seismic velocity model provide evidence for this southward migrating magmatic flare-up mechanism: (1) the volumes of low-velocity zones associated with magmatic reservoirs decreases from north to south across the Puna Plateau, (2) the lowest velocities observed in the core of any reservoir progressively increases from north to south (Fig. 3), (3) the depths (b.s.l.) at which the lowest velocity (center of low-velocity zone) is imaged in each of the five low-velocity zones (IBMB 33 km; IMB 20 km; CGMB 18 km; LMB 16 km; and APMB 11 km) systematically deepens from north to south (Fig. 2). At smaller scales, the minimum velocities we observe within the APMB low-velocity zone are asymmetrically distributed, with the youngest ignimbrite deposits in the APVC (<2 Ma) located above the lowest seismic velocities imaged in the magma reservoir (Fig. 3). This suggests the ~200 km diameter APMB low-velocity zone is internally complex, likely reflecting the episodic and incremental assembly of individual plutonic volumes^47^.

Although our seismic velocity model only reveals the current magmatic state of the crust in the Puna, the unique southward migration of the mechanism responsible for the magmatic flare-up allows us to interpret along-strike variations as different stages in the magmatic evolution of the Puna Plateau. Under this paradigm, the sequence of south to north cross-sections (Fig. 2) illustrates the magmatic evolution in a thermally warming and tectonically widening thickened crust. Starting with a narrow, relatively cool, and thick crust at 28°S, the main crustal magma reservoir is centered at mid-crustal levels beneath the arc and nearly spherical in shape (Fig. 2e). The southern Puna cross-sections (Fig. 2b–d) reveal a wider zone of thickened crust with multiple, sill-like magma reservoirs situated in both the middle and upper crust of the arc and backarc. At the northern end of the Puna, individual upper crustal magma reservoirs have amalgamated into a single, shallow, ~200 km wide sill-like magma reservoir with the slowest observed seismic velocities underlying the area with the most voluminous and long-lived volcanism in the APVC (Fig. 3). These observations are consistent with the multi-stage conceptual model of continental arc evolution inferred from recent numerical simulations of multiple pulsing and dike/diapir intrusions of granitic magma into the continental crust^48^.

**Figure 4:**
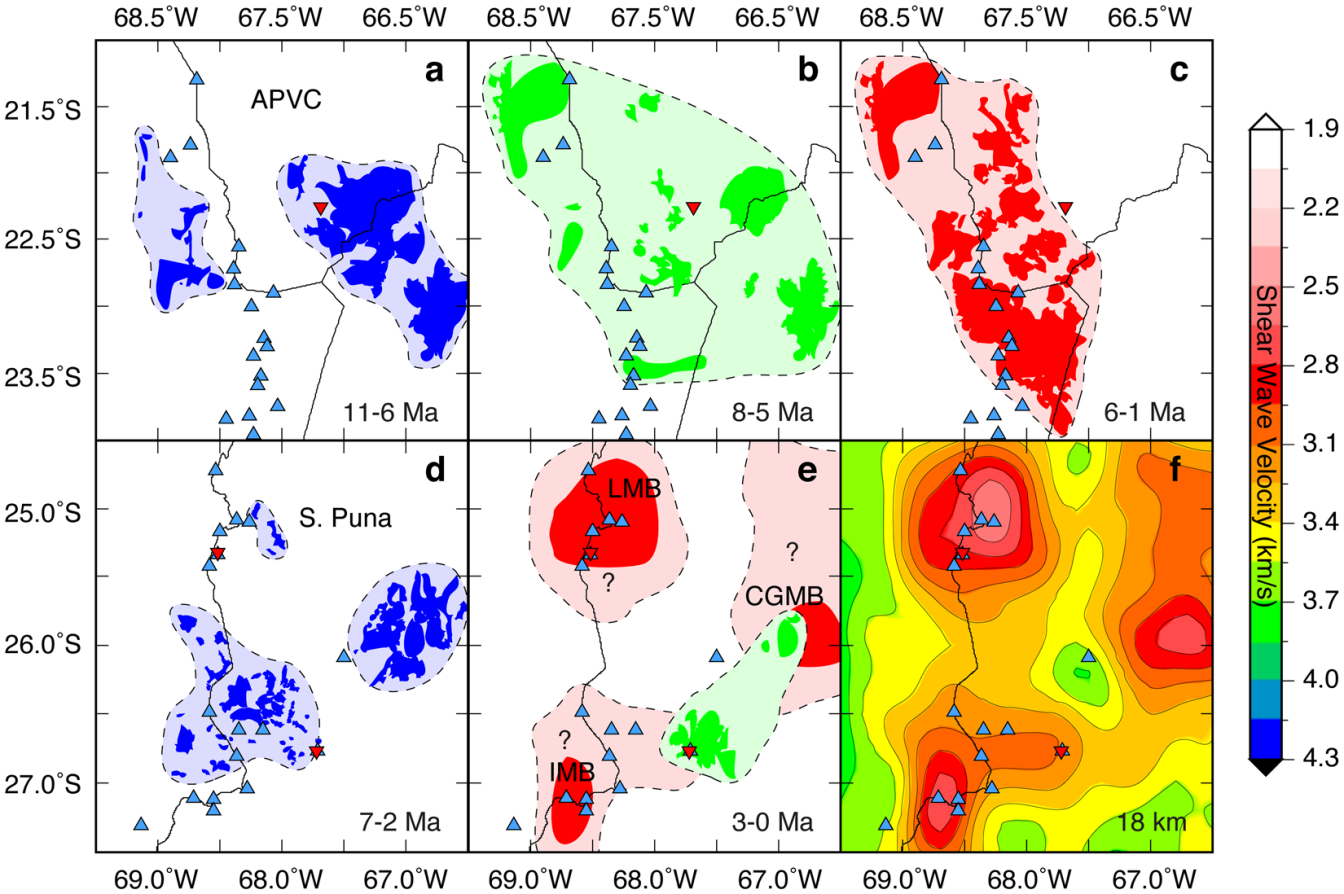
(**a–c**) The spatial-temporal evolution of ignimbrite emplacement (Freymuth *et al*.^11^ and references therein) during the Altiplano-Puna Volcanic Complex (APVC) ignimbrite flare-up episode (11-1 Ma) with blue triangles showing Holocene age volcanism and inverted red triangles show INSAR measured vertical surface deformation centers^28^. Colors in each panel (blue, green, and red) represent the spatial distribution of ignimbrites erupted during that time period only, and appear to correlate with spatial configurations in the southern Puna. (**d–e**) The spatial-temporal evolution of ignimbrite emplacement for the southern Puna. Colors in each panel (blue and green) represent the spatial distribution of ignimbrites erupted during that time period only, and appear to correlate with spatial configurations in the APVC. Red contoured bodies represent the interpreted lateral extent of magma reservoirs inferred from the shear velocity model shown in panel (**f**). (**f**) Horizontal 18 km depth slice through our velocity model showing the lateral extent of interpreted magma reservoirs. The labels LMB (Lazufre Magma Body), CGMB (Cerro Galan Magma Body), and IMB (Incahuasi Magma Body) correspond to imaged magma reservoirs discussed in the text. These plots were made using the Generic Mapping Tool^61^, version 4.5.1 (ftp://ftp.soest.hawaii.edu/gmt).

We evaluate the predictive power of our velocity model to infer the future magmatic evolution of the southern Puna Plateau by subdividing the extremely detailed spatial-temporal record of ignimbrite emplacement (Fig. 1b) mapped across the APVC and southern Puna^13, 14, 44^ into three distinct spatial configurations (Fig. 4). Using the plutonic volumes imaged from our velocity model as a proxy for the spatial distribution of future ignimbrite eruptions (Fig. 4e,f), numerous spatial-temporal similarities are observed between the evolution of the APVC magmatic flare-up and the augmented magmatic history (Fig. 4) inferred for the southern Puna. We therefore suggest the crust in the APVC represents a thermally evolved version of the southern Puna crust. A corollary of this interpretation is that silicic magmatism in the southern Puna will eventually resemble the APVC ignimbrite flare-up episode (Fig. 4).

## Implications for the generation of silicic crust

The presence of multiple voluminous *in-situ* proto-plutonic magmatic bodies in the Puna Plateau indicates that the generation of silicic crust from the influx of mantle-derived melts combined with the reworking of pre-existing crust is volumetrically large. Our large, slow seismic velocity bodies indicate the presence of melt and presumably magmatic hybridization, as seen explicitly in the geochemical signatures beneath at least one of these volcanic centers (Cerro Galan^49^). A recent hypothesis by Cao *et al*.^50^ posited that production of silicic crust in continental arcs is responsible for the global population distribution of zircons, thus indicating that continental arcs play a major role in the generation of silicic crust, at least since 750 Ma. The large, *in-situ* silicic magmatic bodies^51^ imaged in this study directly support the idea that large volumes of silicic material can be generated in the crust at continental arcs. The implications are that the evolution of long-lived continental arcs during the plate tectonic cycle may have substantially contributed to the generation of new continental crust and perhaps more importantly, to the differentiation of a more silicic crust through large-scale crustal modification, leading to the varied crustal compositions unique to Earth in our solar system.

## Methods

The details of the procedures used to obtain this shear-wave velocity model are outlined by previous studies^26, 27, 52^. A brief discussion of the data specific to this study is included in the following sections.

### Ps receiver function data

Teleseismic data from 1296 events > = Mw 5.5 was obtained through IRIS and the GFZ for all available short period and broadband stations in the study area. This involved a total of 424 stations from networks deployed during various time periods spanning 1994–2016. For short period data, instrument responses were removed to boost low frequency amplitudes and obtain teleseismic waveforms similar to the broadband station data. The short period and broadband data were then filtered (bandpass from 0.05–4 Hz) and visually inspected, and any waveforms that did not show a clear P-wave arrival were discarded. High-quality waveforms were then used to compute receiver functions with a Gaussian pulse width of ~1 second (corresponding to a 2.8 Gaussian alpha parameter). The resulting receiver functions were then visually inspected using the FuncLab software package^53^ to ensure only high-quality data and receiver functions were used for further analysis. In total, over 8,000 P-wave receiver functions (out of ~22,000 computed) passed the quality control procedures. Finally, Common Conversion Point (CCP) Stacking Analysis^54^ were created with a bin spacing of 0.1 degrees, a minimum bin width of 0.2 degrees, a vertical bin size of 0.5 km, and a minimum of 10 receiver functions per bin. If <10 receiver functions were present in any bin, the bin width would dilate until at least 10 rays were incorporated or a bin width of 1 degree was reached. Data was migrated to depth using a 1-D layer over half-space velocity model consisting of a 65 km thick crust (Vp = 6.1 km/s, Vp/Vs = 1.74) overlying a mantle with Vp = 7.5 km/s and Vp/Vs 1.78.

### Ambient noise tomography data

The majority of the ambient noise-derived Rayleigh wave dispersion data is presented in Ward *et al*.^52^. Rayleigh wave phase velocities from 13 periods (8, 10, 12, 14, 16, 18, 20, 25, 30, 35, 40, 45, 50) were inverted in this study. Data from the PLUTONS^55^ and PUDEL/SLIP^56, 57^ deployments were incorporated when they became publicly available.

### Joint Inversion

We follow the approach of Delph *et al*.^26^ for the joint inversion of the Common Conversion Point-derived receiver functions and ambient noise tomography data^27^. CCP profiles are migrated back to time using the same velocity model for the depth migration, thus minimizing the effects that an incorrect velocity model will have on the resulting CCP-derived receiver functions. To mitigate the effects of spurious artifacts resulting from CCP Stacking Analysis, the CCP-derived receiver functions were filtered with a Gaussian consisting of a 5.55 alpha parameter, leading to creation of receiver functions with an apparent Gaussian alpha parameter of 2.5 (~1 km vertical resolution). A constant velocity (4.5 km/s) initial model with 1 km thick layers was used for the joint inversion. Thus, all “structure” in the shear-wave velocity model is imposed by the data itself (i.e. no a priori structure in the initial velocity model). We use the joint inversion algorithm of Julià *et al*.^58^ with a damping parameter of 0.5 and dataset weighting of 0.3 (receiver function misfit accounts for 70% of the L2-norm penalty function). For more details on the joint inversion procedures of these two datasets, see Delph *et al*.^26^ and Ward *et al*.^24^.

### Data Availability

The datasets analyzed during the current study are available from the facilities of the IRIS Data Management System, and specifically the IRIS Data Management Center (http://ds.iris.edu/ds/nodes/dmc/) and from the GEOFON Program of GFZ Potsdam (http://geofon.gfz-potsdam.de/). The shear-wave velocity model used in this study will be available from IRIS Data Services Products: Earth Model Collaboration (https://ds.iris.edu/ds/products/emc/).

## Electronic supplementary material

Supplementary Information

## References

[1] Rudnick RL, Gao S (2003). Composition of the continental Crust. Treatise. Geochem..

[2] Hawkesworth CJ (2010). The generation and evolution of the continental crust. J. Geol. Soc. London.

[3] Grove TL, Till CB, Krawczynski MJ (2012). The role of H_2_O in subduction zone magmatism. Annu. Rev. Earth Pl. Sc..

[4] Hawkesworth CJ, Kemp AIS (2006). Evolution of the continental crust. Nature.

[5] Ducea MN, Saleeby JB, Bregantz G (2015). The arhitecture, chemistry and evolution of continental magmatic arcs. Annu. Rev. Earth Pl. Sc..

[6] Annen C, Blundy JD, Sparks RSJ (2006). The genesis of intermediate and silicic magmas in deep crustal hot zones. J. Petrol..

[7] Crisp JA (1984). Rates of magma emplacement and volcanic output. J. Volcanol. Geotherm. Res..

[8] Ducea MN, Paterson SR, DeCelles PG (2015). High-volume magmatic events in subduction systems. Elements.

[9] Schmitt AK (2010). Episodic growth and homogenization of plutonic roots in arc volcanoes from combined U-Th and (U-Th)/He zircon dating. Earth Planet. Sci. Lett..

[10] Allmendinger W, Jordan E, Kay M, Isacks BL (1997). The evolution of the Altiplano-Puna plateau of the Central Andes. Annu. Rev. Earth Planet. Sci..

[11] Freymuth H, Brandmeier M, Wörner G (2015). The origin and crust/mantle mass balance of Central Andean ignimbrite magmatism constrained by oxygen and strontium isotopes and erupted volumes. Contrib. Mineral. Petrol..

[12] de Silva SL (1989). Altiplano-Puna volcanic complex of the central Andes. Geology.

[13] Salisbury M (2011). 40Ar/39Ar chronostratigraphy of Altiplano-Puna volcanic complex ignimbrites reveals the development of a major magmatic province. Geol. Soc. Am. Bull.

[14] Kern JM (2016). Geochronological imaging of an episodically constructed subvolcanic batholith: U-Pb in zircon chronochemistry of the Altiplano-Puna Volcanic Complex of the Central Andes. Geosphere.

[15] Ducea MN, Seclaman AC, Murray KE, Jianu D, Schoenbohm LM (2013). Mantle-drip magmatism beneath the Altiplano-Puna plateau, central Andes. Geology.

[16] Yáñez Ga, Ranero CR, von Huene R, Díaz J (2001). Magnetic anomaly interpretation across the southern central Andes (32°–34°S): the role of the Juan Fernández Ridge in the late Tertiary evolution of the margin. J. Geophys. Res..

[17] Ramos VA, Folguera A (2009). Andean flat-slab subduction through time. Geol. Soc. London Spec. Publ..

[18] Kay SM, Coira B, Viramonte J (1994). Young mafic back-arc volcanic rocks as indicators of continental lithospheric delamination beneath the Argentine Puna Plateau, Central Andes. J. Geophys. Res..

[19] Kay SM, Coira BL, Caffe PJ, Chen CH (2010). Regional chemical diversity, crustal and mantle sources and evolution of central Andean Puna plateau ignimbrites. J. Volcanol. Geoth. Res..

[20] Caffe PJ, Trumbull RB, Coira BL, Romer RL (2002). Petrogenesis of early Neogene magmatism in the Northern Puna: implications for magma genesis and crustal processes in the Central Andean Plateau. Journal of Petrology.

[21] Guzmán S (2011). Petrology of the Luingo caldera (SE margin of the Puna plateau): a middle Miocene window of the arc-back arc configuration. J. Volcanol. Geoth. Res..

[22] Caffe PJ, Trumbull RB, Siebel W (2012). Petrology of the Coyaguayma ignimbrite, northern Puna of Argentina: origin and evolution of a peraluminous high SiO_2_ rhyolite magma. Lithos..

[23] Barton MD (1996). Granitic magmatism and metallogeny of southwestern North America. Geological Society of America Special Papers.

[24] Ward KM, Zandt G, Beck SL, Christensen D, McFarlin H (2014). Seismic imaging of the magmatic underpinnings beneath the Altiplano-Puna volcanic complex from the joint inversion of surface wave dispersion and receiver functions. Earth Planet Sci. Lett..

[25] Obrebski M, Abers GA, Foster A (2015). Magmatic arc structure around Mount Rainier, WA, from the joint inversion of receiver functions and surface wave dispersion. Geochem. Geophys. Geosyst..

[26] Delph JR, Ward KM, Zandt G, Ducea MN, Beck SL (2017). Imaging a magma plumbing system from MASH zone to magma reservoir. Earth Planet Sci. Lett..

[27] Delph JR, Zandt G, Beck SL (2015). A new approach to obtaining a 3D shear wave velocity model of the crust and upper mantle: an application to eastern Turkey. Tectonophysics.

[28] Pritchard ME, Simons M (2004). An InSAR-based survey of volcanic deformation in the central Andes. Geochem. Geophys. Geosyst..

[29] Bachmann O, Miller CF, de Silva SL (2007). The volcanic-plutonic connection as a stage for understanding crustal magmatism. J. Volcanol. Geoth. Res..

[30] Paterson SR, Ducea MN (2015). Arc magmatic tempos: gathering the evidence. Elements.

[31] White SM, Crisp JA, Spera FJ (2006). Long-term volumetric eruption rates and magma budgets. Geochem. Geophys. Geosyst..

[32] Giese P, Scheuber E, Schilling F, Schmitz M, Wigger P (1999). Crustal thickening processes in the Central Andes and the different natures of the Moho-discontinuity. J. S. Am. Earth Sci..

[33] Tierney CR, Schmitt AK, Lovera OM, de Silva SL (2016). Voluminous plutonism during volcanic quiescence revealed by thermochemical modeling of zircon. Geology.

[34] Perkins JP (2016). Surface uplift in the Central Andes driven by growth of the Altiplano Puna Magma Body. Nat Commun..

[35] Jagoutz O, Müntener O, Schmidt MW, Burg J-P (2011). The roles of flux and decompression melting and their respective fractionation lines for continental crust formation: evidence from the Kohistan arc. Earth Planet Sci. Lett..

[36] Ducea MN, Bergantz GW, Crowley JL, Otamendi J (2017). Ultrafast magmatic buildup and diversification to produce continental crust during subduction. Geology.

[37] DeCelles P (2004). Late Jurassic to Eocene evolution of the Cordilleran thrust belt and foreland basin system, western USA. Am. J. Sci..

[38] Chapman JB, Ducea MN, Decelles PG, Profeta L (2015). Tracking changes in crustal thickness during orogenic evolution with Sr/Y: an example from the North American Cordillera. Geology.

[39] Best MG, Christiansen EH, de Silva SL, Lipman PW (2016). Slab- rollback ignimbrite flareups in the southern Great Basin and other Cenozoic American arcs: a distinct style of arc volcanism. Geosphere.

[40] Howard KA (2011). Episodic growth of a Late Cretaceous and Paleogene intrusive complex of pegmatitic leucogranite, Ruby Mountains core complex, Nevada, USA. Geosphere.

[41] Christensen N (1996). Poisson’s ratio and crustal seismology. J. Geophys. Res..

[42] Pritchard ME, Gregg PM (2016). Geophysical evidence for silicic crustal melt in the continents: where, what kind, and how much?. Elements.

[43] Folkes C (2011). A reappraisal of the stratigraphy and volcanology of the Cerro Galán volcanic system, NW Argentina. Bultin of Volcanology.

[44] Kay SM, Coira BL (2009). Shallowing and steepening subduction zones, continental lithospheric loss, magmatism, and crustal flow under the Central Andean Altiplano-Puna Plateau.

[45] Beck SL, Zandt G, Ward KM, Scire A (2015). Multiple styles and scales of lithospheric foundering beneath the Puna Plateau, central Andes. Mem. Geol. Soc. Amer..

[46] Yonkee WA, Weil AB (2015). Tectonic evolution of the Sevier and Laramide belts within the North American Cordillera orogenic system. Earth Sci. Rev..

[47] de Silva SL, Gosnold WD (2007). Episodic construction of batholiths: insights from the spatiotemporal development of an ignimbrite flare-up. J. Volcanol. Geoth. Res..

[48] Cao W, Kaus BJP, Paterson S (2016). Intrusion of granitic magma into the continental crust facilitated by magma pulsing and dike–diapir interactions: numerical simulations. Tectonics.

[49] Kay SM, Coira B, Wörner G, Kay RW, Singer BS (2011). Geochemical, isotopic and single crystal 40Ar/39Ar age constraints on the evolution of the Cerro Galán ignimbrites. Bull. Volcanol..

[50] Cao W, Lee CTA, Lackey JS (2017). Episodic nature of continental arc activity since 750 Ma: A global compilation. Earth Planet Sci. Lett..

[51] Farner MJ, Lee CA (2017). Effects of crustal thickness on magmatic differentiation in subduction zone volcanism: A global study. Earth Planet. Sci. Lett..

[52] Ward KM (2013). Ambient noise tomography across the central Andes. Geophys. J. Int..

[53] Eagar KC, Fouch MJ (2012). FuncLab: A MATLAB interactive toolbox for handling receiver Function datasets. Seismological Research Letters.

[54] Dueker KG, Sheehan AF (1997). Mantle discontinuity structure from midpoint stacks of converted P to S waves across the Yellowstone hotspot track. J. Geophys. Res..

[55] West, M. & Christensen, D. Investigating the relationship between pluton growth and volcanism at two active intrusions in the central Andes. *International Federation of Digital Seismographic Networks* (2010).

[56] Heit, B., Yuan, X., Kind, R. & Günter, A. Lithospheric dynamics in the southernmost Andean platea. *Deutsches Geo Forschung Zentrum GFZ* (2007).

[57] Sandvol, E. & Brown, L. SLIP—Seismic lithospheric imaging of the Puna Plateau, *International Federation of Digital Seismograph Networks* (2007).

[58] Julià J, Ammon CJ, Herrmann RB, Correig AM (2000). Joint inversion of receiver function and surface wave dispersion observations. Geophys. J. Int..

[59] Gioncada A (2010). Pliocene intraplate-type volcanism in the Andean foreland at 26 10°S, 64 40°W (NW Argentina): implications for magmatic and structural evolution of the Central Andes. Lithos.

[60] Bonvalot, S. *et al*. World Gravity Map. *Bureau Gravimetrique International* (BGI) (CGMW-BGI-CNES-IRD Ed., Paris) (2012).

[61] Wessel P, Smith WHF, Scharroo R, Luis JF, Wobbe F (2013). Generic mapping tools: improved version released. EOS Trans. AGU.

[62] Tassara A, Echaurren A (2012). Anatomy of the Andean subduction zone: three-dimensional density model upgraded and compared against global-scale models. Geophys. J. Int..

