# Supplementary material for: Magmatic evolution of a Cordilleran flare-up and its role in the creation of silicic crust
Source: Sci Rep. 2017 Aug 22;7:9047. doi: 10.1038/s41598-017-09015-5 (PMC5567344; doi:10.1038/s41598-017-09015-5)
Supplement: Supplementary file 1 — Supplementary Information [file 41598_2017_9015_MOESM1_ESM.pdf]

Magmatic evolution of a Cordilleran flare-up and its role in the creation of silicic crust

Kevin M. Ward[1,3], Jonathan R. Delph[2,3], George Zandt[3], Susan L. Beck[3], and Mihai N. Ducea[3,4]

[1]*Department of Geology and Geophysics, University of Utah, Salt Lake City, Utah 85721, USA*

[2]*Department of Earth Science, Rice University, Houston, Texas 77005, USA*

[3]*Department of Geosciences, University of Arizona, Tucson, Arizona 85721, USA*

[4]*Faculty of Geology and Geophysics, University of Bucharest, Bucharest, 010041, Romania*

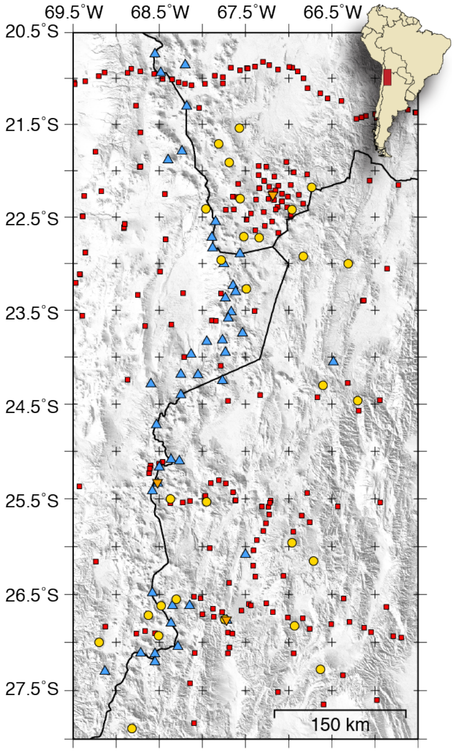

Supplementary Figure 1. A map of our study area with stations used to calculate receiver functions plotted as red squares. This study combines data used in two smaller study areas from Ward et al.[1] and Delph et al.[2] into one larger tomography study. Surface wave data encompasses this study area (extends well beyond this study area) and is from Ward et al.[3]. Blue triangles show Holocene age volcanism of the Central Volcanic Zone (CVZ), gold circles show known ignimbrite eruption calderas[4], inverted orange triangles show INSAR measured vertical surface deformation centers[5]. This plot was made using the Generic Mapping Tool[6], version 4.5.1 (ftp://ftp.soest.hawaii.edu/gmt).

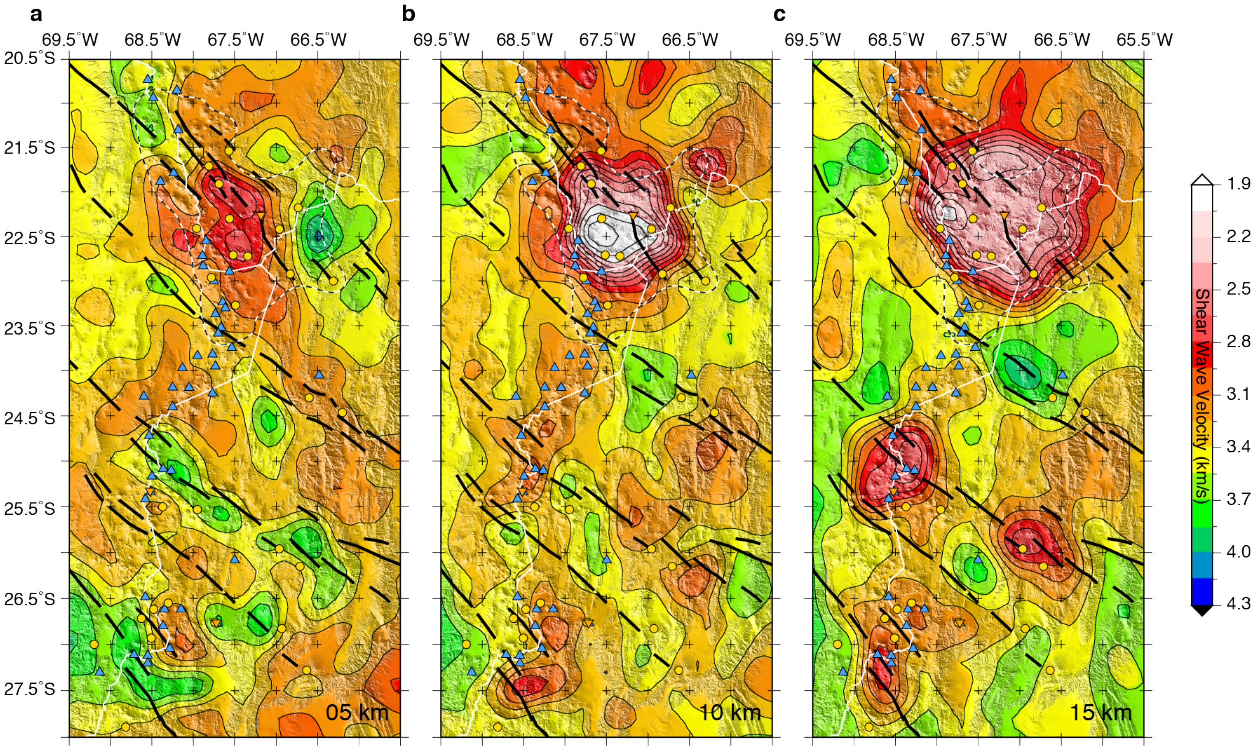

Supplementary Figure 2. a-c: Horizontal depth slices through our shear-wave velocity model for 05, 10, and 15 km depth below sea level. The Altiplano-Puna Volcanic Complex (APVC) is show as a black dashed line with white fill. Blue triangles show Holocene age volcanism of the Central Volcanic Zone (CVZ), gold circles show known ignimbrite eruption calderas[4], inverted orange triangles show INSAR measured vertical surface deformation centers[5], and black lines show the location of transverse lineaments[7]. These plots were made using the Generic Mapping Tool[6], version 4.5.1 (ftp://ftp.soest.hawaii.edu/gmt).

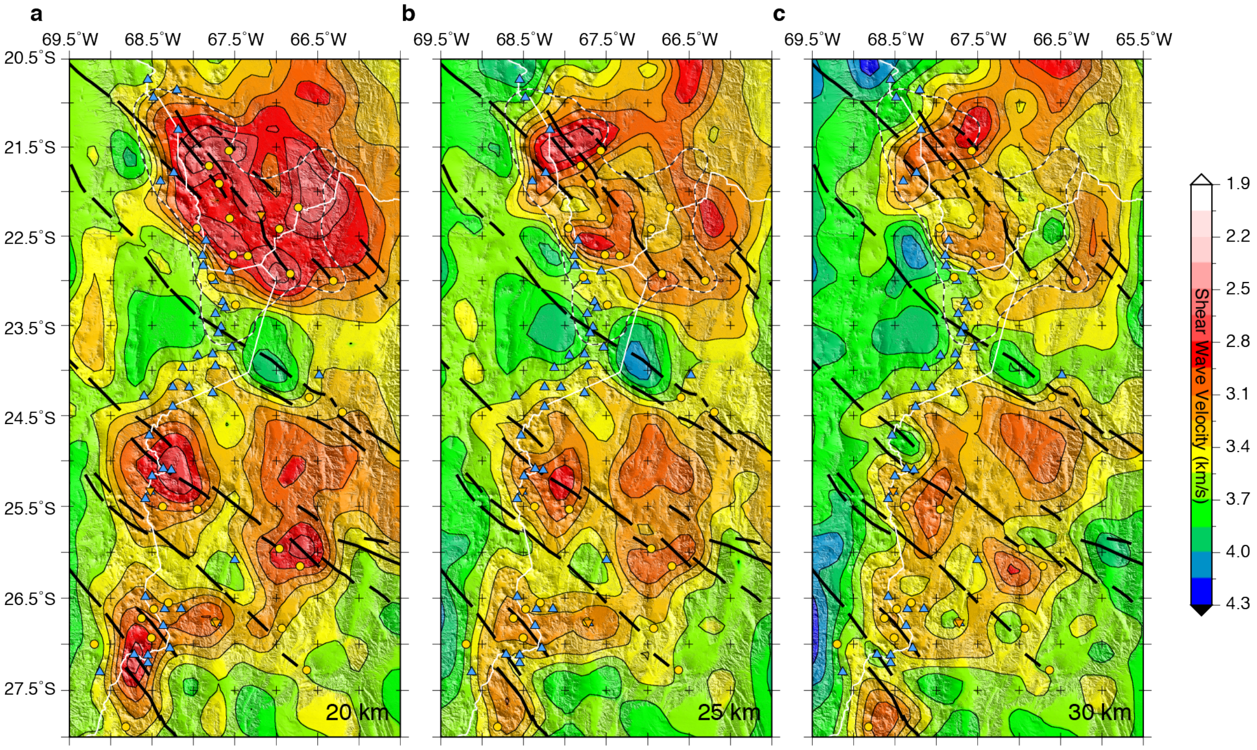

Supplementary Figure 3. a-c: Horizontal depth slices through our shear-wave velocity model for 20, 25, and 30 km depth below sea level. The Altiplano-Puna Volcanic Complex (APVC) is show as a black dashed line with white fill. Blue triangles show Holocene age volcanism of the Central Volcanic Zone (CVZ), gold circles show known ignimbrite eruption calderas[4], inverted orange triangles show INSAR measured vertical surface deformation centers[5], and black lines show the location of transverse lineaments[7]. These plots were made using the Generic Mapping Tool[6], version 4.5.1 (ftp://ftp.soest.hawaii.edu/gmt).

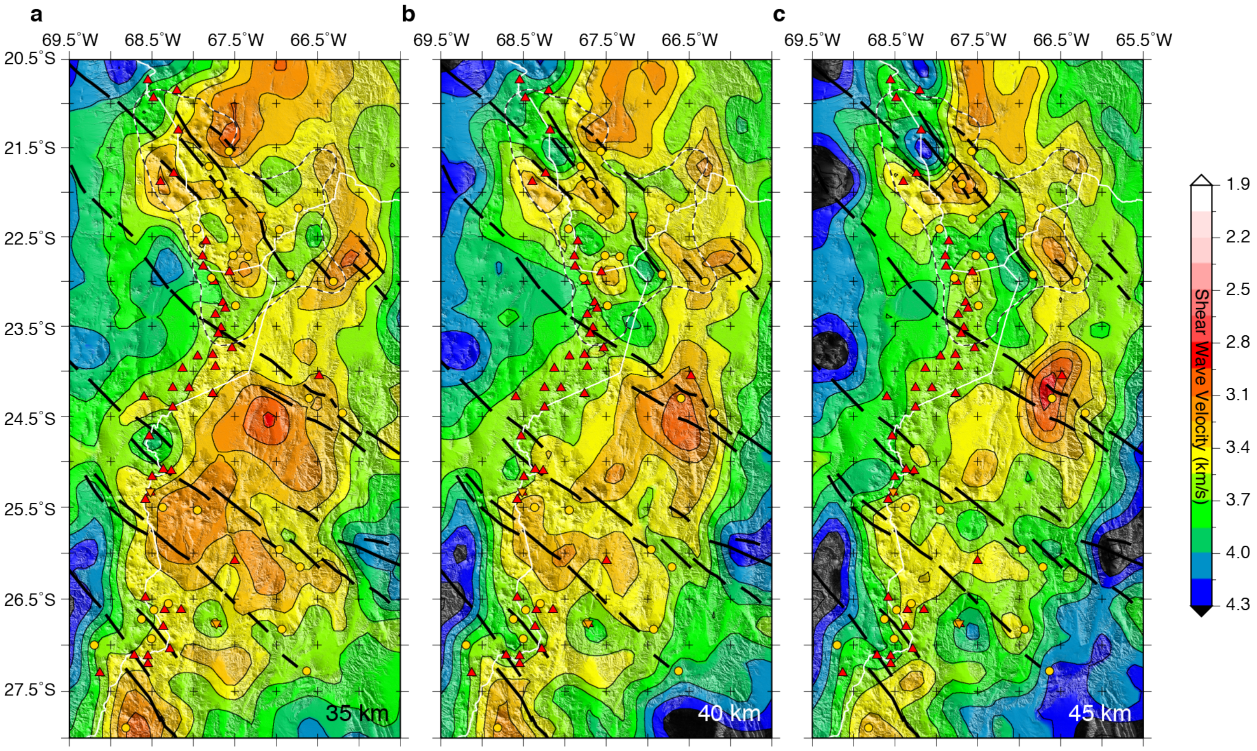

Supplementary Figure 4. a-c: Horizontal depth slices through our shear-wave velocity model for 35, 40, and 45 km depth below sea level. The Altiplano-Puna Volcanic Complex (APVC) is show as a black dashed line with white fill. Red triangles show Holocene age volcanism of the Central Volcanic Zone (CVZ), gold circles show known ignimbrite eruption calderas[4], inverted orange triangles show INSAR measured vertical surface deformation centers[5], and black lines show the location of transverse lineaments[7]. These plots were made using the Generic Mapping Tool[6], version 4.5.1 (ftp://ftp.soest.hawaii.edu/gmt).

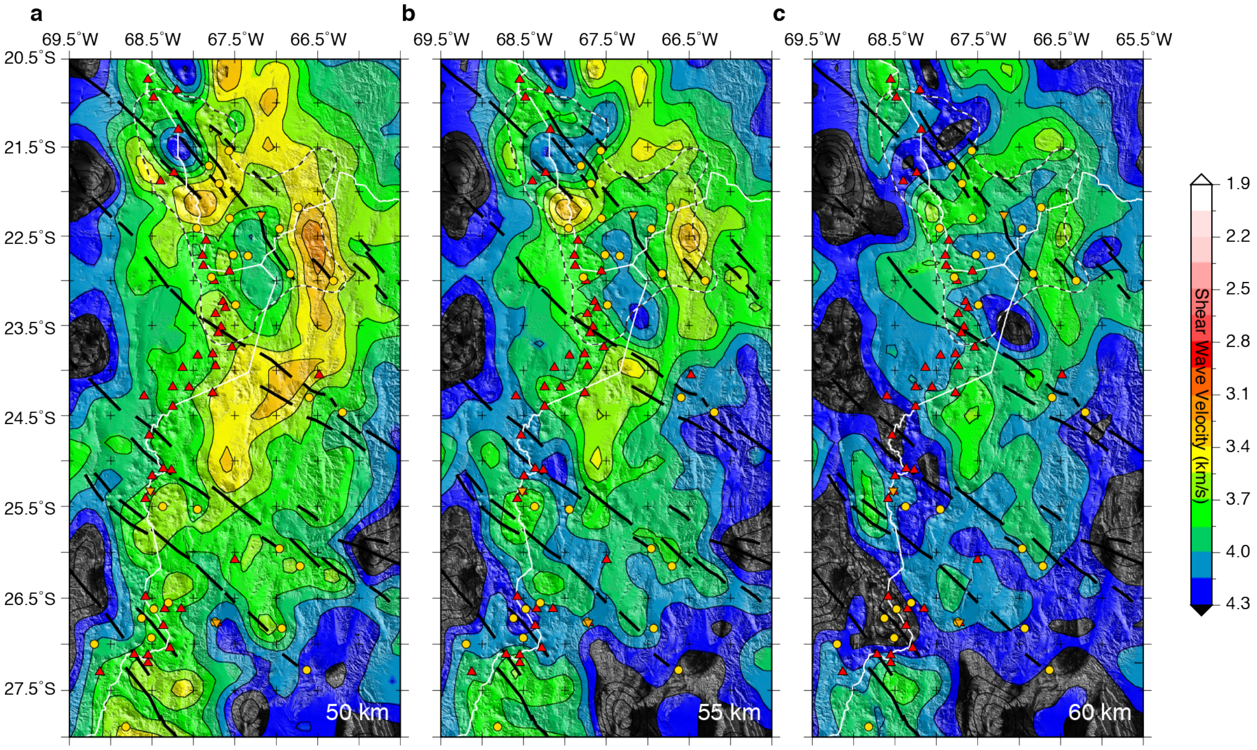

Supplementary Figure 5. a-c: Horizontal depth slices through our shear-wave velocity model for 50, 55, and 60 km depth below sea level. The Altiplano-Puna Volcanic Complex (APVC) is show as a black dashed line with white fill. Red triangles show Holocene age volcanism of the Central Volcanic Zone (CVZ), gold circles show known ignimbrite eruption calderas[4], inverted orange triangles show INSAR measured vertical surface deformation centers[5], and black lines show the location of transverse lineaments[7]. These plots were made using the Generic Mapping Tool[6], version 4.5.1 (ftp://ftp.soest.hawaii.edu/gmt).

References

1. Ward, K. M., Zandt, G., Beck, S. L., Christensen, D. & McFarlin, H. Seismic imaging of the magmatic underpinnings beneath the Altiplano-Puna volcanic complex from the joint inversion of surface wave dispersion and receiver functions. *Earth Planet Sci. Lett.* **404,** 43-53 (2014).
2. Delph, J. R., Ward, K. M., Zandt, G., Ducea, M. N., & Beck, S. L. Imaging a magma plumbing system from MASH zone to magma reservoir. *Earth Planet Sci. Lett.* **457,** 313-324 (2017).
3. Ward, K. M. et al. Ambient noise tomography across the central Andes. *Geophys. J. Int.* **194,** 1559-1573 (2013).
4. Freymuth, H., Brandmeier, M., Wörner, G. The origin and crust/mantle mass balance of Central Andean ignimbrite magmatism constrained by oxygen and strontium isotopes and erupted volumes. *Contrib. Mineral. Petrol.* **169,** 1-24 (2015).
5. Pritchard, M. E. & Simons, M. An InSAR-based survey of volcanic deformation in the central Andes. *Geochem. Geophys. Geosyst.* **5,** 1-42 (2004).
6. Wessel, P., Smith, W. H. F., Scharroo, R., Luis, J. F. & Wobbe, F. Generic mapping tools: improved version released. EOS Trans. AGU **94,** 409-410 (2013).
7. Gioncada, A. et al. Pliocene intraplate-type volcanism in the Andean foreland at 26 10°S, 64 40°W (NW Argentina): implications for magmatic and structural evolution of the Central Andes. *Lithos* **2,** 153-171 (2010).
